# Social Influence in Adolescent Decision-Making: A Formal Framework

**DOI:** 10.3389/fpsyg.2019.01915

**Published:** 2019-08-29

**Authors:** Simon Ciranka, Wouter van den Bos

**Affiliations:** ^1^Center for Adaptive Rationality, Max Planck Institute for Human Development, Berlin, Germany; ^2^Max Planck UCL Centre for Computational Psychiatry and Ageing Research, Berlin, Germany; ^3^Department of Developmental Psychology, University of Amsterdam, Amsterdam, Netherlands

**Keywords:** adolescence, decision-making, social influence, risk-taking, expected utility, computational modeling, hierarchical bayes

## Abstract

Adolescence is a period of life during which peers play a pivotal role in decision-making. The narrative of social influence during adolescence often revolves around risky and maladaptive decisions, like driving under the influence, and using illegal substances (Steinberg, 2005). However, research has also shown that social influence can lead to increased prosocial behaviors (Van Hoorn et al., 2017) and a reduction in risk-taking (Braams et al., 2019). While many studies support the notion that adolescents are more sensitive to peer influence than children or adults, the developmental processes that underlie this sensitivity remain poorly understood. We argue that one important reason for this lack of understanding is the absence of precisely formulated models. To make a first step toward formal models of social influence during adolescence, we first identify three prominent verbal models of social influence in the literature: (1) social motivation, (2) reward sensitivity, and (3) distraction. We then illustrate how these can be translated into formal models, and how such formal models can inform experimental design and help identify developmental processes. Finally, by applying our formal models to existing datasets, we demonstrate the usefulness of formalization by synthesizing different studies with seemingly disparate results. We conclude with a discussion on how formal modeling can be utilized to better investigate the development of peer influence in adolescence.

## Introduction

Peers impact almost all aspects of adolescent lives, from the more trivial, such as taste in music and clothing, to the more serious, such as the use of illicit drugs or engaging in unprotected sex (Steinberg, 2008). These latter, riskier, choices may carry life-long consequences for the adolescent and bring significant cost to society. It is empirically well established that the presence of peers influences risky behavior in adolescence (Gardner and Steinberg, 2005; Chein et al., 2011; Pfeifer et al., 2011; Smith et al., 2014), but the underlying developmental processes remain poorly understood. Understanding these processes, however, is important for at least two reasons. First, empowering adolescents to become more competent decision-makers will be more effective if we succeed at tailoring interventions to their developmental affordances. Second, we can only identify these affordances if we succeed at linking adolescent neuronal and cognitive development with adolescent behavior across different social contexts.

Here we argue that this link cannot be made without formal models of adolescent peer influence. In this article we thus aim to take the first steps toward a quantitative and testable framework of adolescent social influence. Adolescence is marked by several developmental changes which offer multiple biological explanations of social influence on adolescent decision making. We refer to the current theoretical perspectives of these changes as “verbal models.” Verbal models are distinct from formal models in that they do not make quantitative predictions. In order to establish formal models that do make quantitative predictions, we first review existing verbal models and the associated empirical findings about social influence in adolescents, focusing on risky decision-making. We identify three verbal models of social influence which can be subject to developmental change; these are then formalized by grounding them in expected utility theory. Next, we show that our formal models can reliably be recovered and therefore can be used to compare hypotheses via quantitative model comparison. Finally, we fit these models to existing data and reveal previously overlooked patterns of peer influence. We conclude with a discussion on how the specificity provided by this formal approach contributes to a deeper understanding of the developmental processes behind social influence.

### Social Influence on Adolescent Decision-Making

We identify three main families of verbal models in the existing literature, hereafter named as follows: (i) social motivation model, (ii) reward sensitivity model, and (iii) distraction model. These three models focus on two distinct neurodevelopmental explanations of altered decision-making during adolescence. *Social motivation* verbal models stress the importance of the developing “social brain.” The other two verbal models (*reward sensitivity* and *distraction*) both emphasize the relatively slow maturation of cognitive control systems. Previous works that fall into the *reward sensitivity* family of verbal models often refer to it as “dual-systems” models, as they also stress the relatively fast maturation of reward-processing brain regions and explain adolescent behavior with the maturational imbalance between reward processing and cognitive control brain regions (Casey et al., 2008; Steinberg, 2008; Geier et al., 2010; Shulman et al., 2016). By contrast, the *distraction* model has a single focus on the development of cognitive control. Our subsequent review of the existing experimental evidence shows that all three of these families of verbal models are currently equally well supported in the literature, even though each model provides a different explanation for similar observations.

#### Verbal Models: Social Motivation

The first verbal model we consider states that adolescents have increased social motivation. Demonstrating risky behavior, or conforming to behavior of the peer group, are considered ways to reach these social goals. In other words, social motivation models assume that during adolescence there are situations where a high social value is attributed to displaying risky behavior (Crone and Dahl, 2012; Ruff and Fehr, 2014) which is independent from the non-social value of the outcome (e.g., money).

#### Verbal Models: Reward Sensitivity

The verbal reward sensitivity model is based on research which suggests that adolescence is the time where fast maturation of reward processing brain systems coincides with relatively slow maturation of cognitive control systems. According to the reward sensitivity model, the biological imbalance between these two systems gives rise to risky adolescent decision-making (Casey et al., 2008; Ernst et al., 2015; Shulman et al., 2016). Here we will not address the debate concerning the validity (Pfeifer and Allen, 2016) or the different variants of these models (Casey et al., 2008; Steinberg, 2008; Larsen and Luna, 2018). Instead, we focus on the element that is suggested to be most relevant for understanding developmental changes in peer influence: reward sensitivity. Reward sensitivity states that social influence has such dramatic effects on adolescent risk-taking because a social context “may sensitize the incentive processing system to respond to cues signaling the potential rewards of risky behavior” (Chein et al., 2011, p. 2). Indeed, Chein et al. (2011) showed that while being observed during a risk-taking task, brain regions related to reward processing were more active in adolescents than in adults. This was interpreted as evidence for a reward sensitivity model as it suggests that, in adolescents, the social context itself leads to changes in the processing of rewards in general.

#### Verbal Models: Distraction

The relatively slow maturation of cognitive control brain regions forms the basis of a third verbal model that we call “distraction model.” Here, maturational imbalance and arousal is not only specifically associated with altered representations of reward but more generally with poor self-control and diminishing cognitive skills in emotionally salient situations (Dumontheil, 2016). This lack of self-control can lead adolescents to show more erratic or distracted behaviors in a social as compared to a solitary context. The distraction model does not assume any changes in value computation, but rather suggests that behavioral changes are due to stochasticity in the decision process.

Social motivation, reward sensitivity and distraction models do not assume mutually exclusive processes. Although it is plausible that the defining processes emphasized in each of these models simultaneously impact peer influence, it is important to examine which are most relevant in a particular context.

This is essential because different models provide different footholds for interventions. For example: if adolescent risk-taking is subject to social motivation it can be fruitful to provide other, less risky, means to acquire social status for instance by using meaningful roles interventions (Ellis et al., 2016, see also: Yeager et al., 2018). Adolescent reward sensitivity suggests it is useful to prohibit teens from gathering in risky situations. For instance, many states in the United States and Canada prohibit teenage drivers from taking other teenage passengers along. Distraction suggests that training in mindfulness and meditation are a good prospect for increasing desirable behaviors in adolescence (Kuyken et al., 2013). These implications for interventions underscore how crucial it is to comprehend the most relevant determinants of adolescent behavior in a given context. We therefore inspect experimental work which manipulated aspects of social contexts with respect to the three verbal models of adolescent social influence: (i) social motivation, (ii) reward sensitivity, and (iii) distraction.

### Seeing and Being Seen – Empirical Studies of Social Influence

Despite the complexity of social exchange, studies investigating social influence can be roughly divided into two types of situations: those where the participant observes others and those where the participant is being observed. In the light of this distinction, we review experimental studies about peer influence in adolescent risky decision-making.

#### Observing Others

When uncertain of what to do, observing the behavior of others can help with making a decision. Monetary lotteries are often used as an experimental setting with uncertain prospects, wherein the effect of observing the behavior of others can be investigated. In such experiments, participants observe others’ previous decisions (Blankenstein et al., 2016; Reiter et al., 2019) or receive explicit advice (Haddad et al., 2014) while making private decisions. These studies suggest that the impact of social information is greatest in early to mid-adolescence and then declines with age. Notably, in a recent study, adolescents were influenced more by safe than by risky advice (Braams et al., 2019). However, currently evidence seems most in line with models that emphasize social motivation, as an increase in safe decisions is not predicted by reward sensitivity models. A small increase of participant safe choices in studies such as Braams et al. (2019) however, could also be attributed to a greater distraction during adolescence. Notably, none of these studies provided adolescents with information about the outcomes of others’ decisions. In real life, such outcomes are observable; there is evidence that observing others’ risky real-world behaviors, such as smoking or drug use, increases the likelihood of adolescents to adopt these behaviors themselves (Clark and Lohéac, 2007; Liu et al., 2017). This can reasonably be explained using social motivation models, if adolescents anticipate peer approval. It can also be explained with reward sensitivity models when assuming that the rewarding properties of risky behaviors (smoking) themselves become subjectively more rewarding in this social context.

In sum, experimental results from paradigms in which participants observe the choices of others are sometimes more consistent with the social motivation model, and sometimes more consistent with reward sensitivity. Paradigms designed for testing distraction models when observing others are underrepresented, so their pertinence here cannot yet be sufficiently evaluated. As such, which verbal model family best accounts for adolescent behavior when they observe others remains unclear.

#### Being Observed

When a decision maker is observed by another individual, risk-taking also sends a social signal to the observer (Baker and Maner, 2009). For instance, adolescents can show how “cool” they are by taking extreme risks, or signal that they are or want to be part of a group by mimicking its members’ risk-taking behavior. Thus, if adolescent behavior in peer contexts is sending a social signal to their peers, their beliefs about the risk-norms of observing peers should impact their behavior. In line with this, one study found that exposing teenagers to risk-accepting peers increased their risky driving while exposure to a risk-averse peers did not (Shepherd et al., 2011). Further, there is evidence that risk perception and understanding of social norms are important predictors of adolescent risky driving (Carter et al., 2014). Social motivation models can therefore explain increased risk-taking in paradigms when participants are being observed.

However, even without assuming complex social motivation, behavior change in a social context was traditionally explained with social facilitation theory (Zajonc, 1965), which foreshadowed both reward sensitivity, and distraction models by one principled observation: Being observed induces arousal.

The reward sensitivity model suggests that arousal leads to altered reward processing, making risk-taking more appealing. Indeed, most developmental studies of how being observed impacts risk-taking report an increase in the number of risky choices made by adolescents in social contexts (Gardner and Steinberg, 2005; Chein et al., 2011; Smith et al., 2014; Somerville et al., 2018). In the context of social facilitation theory this increase in risk-taking can be seen either as facilitation, for example by increasing explorative behaviors and socially acceptable risk-taking, or impediment, when the risks are illegal and dangerous (Duell and Steinberg, 2019). In one remarkable neuroimaging study along these lines (Chein et al., 2011), found evidence for the reward sensitivity model. The presence of another person increased activity in the ventral striatum when adolescents received rewards, as compared to a solitary reward condition. This was true for adolescents but not for adults.

However, in another variant of social facilitation theory (Sanders et al., 1978), social arousal is thought to result in distraction from the task at hand, thus mostly resulting in detrimental or sub-optimal behavior. In fact, there is evidence that arousal leads to decreased cognitive control, which results in more distracted behavior in decision-making tasks (Starcke and Brand, 2012). There is also evidence that distraction accounts for typical adolescent behavior in some experimental paradigms. For instance, Dumontheil et al. (2016) demonstrated reduced reasoning abilities in adolescents when monitored by peers. Similarly, another study found that adolescents who showed poor conflict monitoring in an emotionally arousing Stroop task also turned out to be risky drivers in a driving simulator (Botdorf et al., 2017).

Consequently, changes in risky choice while being observed could be the result of the motivation for social signaling, of arousal-based reward sensitive decisions, or distraction, and each of these three processes possibly has a different developmental trajectory. Merely observing an increase in risky decisions in adolescents seems insufficient to specify which underlying psychological process is most relevant.

In sum, different studies have emphasized different models and found results in favor of each. This holds for paradigms when adolescents are observing others and even more for paradigms in which they are observed. These mixed results may be due to the fact that each study has used different experimental paradigms with large variations of the key variables (e.g., known risk vs. uncertainty, best friend vs. unknown peer) and most studies do not directly compare different social contexts in order to identify if they are subject to different psychological processes (but see Somerville et al., 2018). Another reason for the diversity of experimental findings, which can also be attributed to variations in key variables, is that studies likely differ in their affective content. For instance, the affective content of a study on social influence which only uses information about choices of strangers who are not currently present is fundamentally different from a study wherein social influence is examined by looking at changes in behavior in the presence of a close friend. The distinction between affectively “hot” and “cold” contexts is a useful heuristic to understand adolescent risk-taking. There is evidence that adolescents make more risky choices in “hot” contexts. Notably, reward sensitivity and distraction models explain behavior change via affect (arousal) as well (Blakemore and Robbins, 2012; Rosenbaum et al., 2018). In order to comprehend adolescent socio-emotional development, we need to better understand how affect and social processing interact and impact each other. We argue that the specificity provided by formal modeling might help to disentangle these important components in developmental research, similar to the field of computational psychiatry (Montague et al., 2012; Huys et al., 2015; Jolly and Chang, 2018).

However, before further elaborating on the benefits of formal models in developmental research we first want to pay credit to the neuroscience of adolescent development. Neuroimaging studies may provide better clues to what extent different processes underlie behavior. In addition, it may be possible to generate more specific hypotheses about which psychological processes are involved based on the localization of neural activation.

### Social Influence and Brain Development

Most verbal models of adolescent social influence are inspired by recent findings from developmental neuroimaging. Here we will review some of those findings and indicate to what extent they support existing models. Given that neural activation is a more direct reflection of the processes underlying behavior, neuroimaging may be instrumental to identify which process is most relevant in which context.

Adolescent social motivation models are supported by findings about the development of a network of brain regions associated with social cognition. This network, sometimes subsumed as the “social brain,” continues to develop during adolescence (Mills et al., 2014). The most prominent regions of this network are the temporo parietal junction (TPJ), the posterior superior temporal sulcus (pSTS), the anterior temporal cortex (ATC), and the medial prefrontal cortex (mPFC). When reasoning about others, the social network seems more active in adolescents than in adults or children (van den Bos et al., 2011). Further, in a study by Somerville et al. (2013) observed by others resulted in increased mPFC activity in adolescents. However, activity in these regions is not unique to social processing. For instance the same study found an adolescent increase in connectivity of the mPFC with striatal brain regions, which are relevant for processing rewards. Further, the mPFC itself is also involved in basic reward processing (Harris et al., 2007; Silverman et al., 2015). Taken together, the increased mPFC activity when being observed can also be interpreted as supporting the reward sensitivity model.

Neural correlates of the role of adolescent reward sensitivity in non-social contexts were recently examined in a meta-analysis (Silverman et al., 2015). This study estimated an increased likelihood of activation in adolescents within a broad range of regions associated with reward processing. These comprise the ventral and dorsal striatum, subcallosal cortex, insula, and amygdala as well as the anterior cingulate cortex (ACC), the posterior cingulate cortex (PCC), and the paracingulate region and the medial prefrontal cortex (mPFC). One study found increased activity in the ventral striatum when adolescents where taking risks in a social but not in a solitary context, whereas this activity difference was not found in adults (Chein et al., 2011). These results are evidence in favor of the reward sensitivity model, but there are multiple possible interpretations. For instance, increased reward related neural activity could either be the result of altered reward perception or of an orthogonal, social value of conforming to a norm. Both social and non-social value is represented in the striatum (Ruff and Fehr, 2014); both mechanisms can lead to more risky behavior in certain tasks.

Distraction models emphasize the development of the lateral prefrontal cortex (lPFC) and the inter parietal sulcus, which make up the main regions of the cognitive control network. Studies based on the distraction model consistently found increased IPS activation during cognitive control in adolescents, whereas lPFC findings were mixed (Dumontheil, 2016). One study investigating the effects of social context on neural processing while performing a relational reasoning task found that adolescents recruited this cognitive control network more strongly than adults when an audience was present, while performance changed in a similar magnitude for both age groups (Dumontheil et al., 2016). This result also allows for multiple interpretations. Adolescents may be more distracted, but on the other hand it may also be that they exert more control to counteract their distraction, and thus stay on par with adult’s behavior. The fact that they exert more control could potentially be the result of an increased motivation to perform well while observed by others.

In summation, we have seen that all verbal models are supported by neuroimaging research. Different models emphasize the development of different brain networks, but these networks often overlap with respect to functional and structural components. As long as a one-to-one mapping between cognitive and neural processes is not given, it is not justifiable to make the reverse inference about the presence or absence of a cognitive process purely on the basis of observed, or unobserved neural activity (Poldrack, 2006, 2011).

We do not wish to discredit the existing studies on neural correlates of adolescent peer influence; On the contrary, we believe that these are excellent and well-designed neuroimaging studies. In combination with appropriate experimental control conditions, reverse inference is valid and insightful (Hutzler, 2014). However, experimentally isolating a cognitive process becomes exponentially difficult when the processes in question increase in complexity. Different attempts have been suggested to attenuate the issue, such as large scale brain decoding (Poldrack, 2011; Yarkoni et al., 2011), using functional localizers (Saxe et al., 2006), and formal modeling (Marr and Poggio, 1976; Montague et al., 2012; Stephan et al., 2015; van den Bos and Eppinger, 2016; Hauser et al., 2018). None of these strategies will completely solve the problem of reverse inference, however, each may increase our confidence in reliably identifying the neural correlates of a particular cognitive processes. This article is motivated by the advantage of formal models; in what follows, we will illustrate how verbal models of social influence in adolescence might be translated into formal ones.

## Formal Models of Social Influence

Here we demonstrate how the three verbal models about adolescent socioemotional development which we introduced earlier can be formalized as variations of expected utility models. We then show that model comparison can be used to infer underlying social mechanisms. The rationale behind formal modeling of cognition is that in order to identify if behavior is consistent with a proposed cognitive process, we need to formulate algorithms that represent the process mathematically. Comparing the behavior of the algorithms with actual behavior observed in participants can subsequently be used to quantify support for the hypothesis which is represented by the algorithm. In this section, we aim to translate verbal models of adolescent development into formal ones. However, current models often lack the details required in order to be directly translated into formal models. To formalize the models, we have therefore made several assumptions rooted in expected utility theory. The model space that we present here is not exhaustive. Nevertheless, the current framework illustrates how formal modeling can be used in developmental science, and provides a strong starting point for developing more elaborate models. More importantly, it enables precise discussions on which models are favored by existing experimental data. To formalize models of adolescent decision making First, we address how risk seeking behavior is understood within the expected utility framework in order to familiarize the reader with its’ assumptions. Then we extend these models with parameters that can be read as social sensitivity, reward sensitivity, and distraction. This finally enables us to test models of adolescent development against one another, even within the same experiment.

### Expected Utility

The first assumption of expected utility theory is that people have a subjective experience of objective rewards. For instance, the first dollar someone ever earns is worth more to them than the hundredth. The change in wealth from nothing to $1 feels different from the change in wealth from $99 to $100. This transformation of objectively equal values ($1 in both cases) into a subjective utility is often modeled by a power function borrowed from psychophysics (Helmholtz, 1896), where it is used to describe the non-linear relationship between subjective psychological experience of a stimulus intensity and the objective physical intensity of the stimulus:

(1) $$U=V^{\rho },$$

**FIGURE 1:**
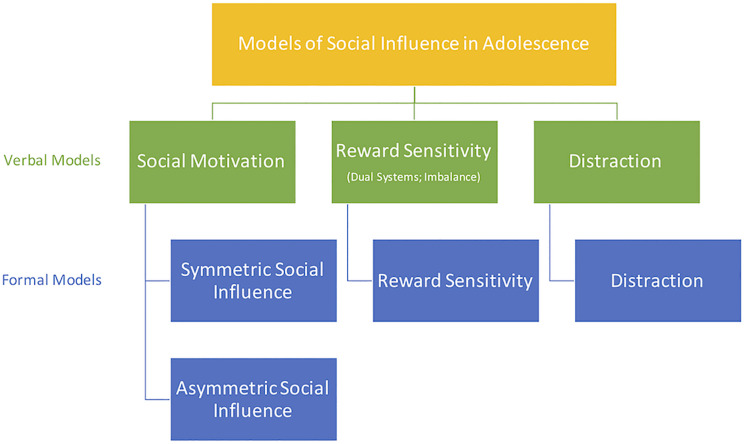
Verbal models of social influence during adolescence, and how they map to our taxonomy of formal models.

Where *V* denotes the objective value of a reward and *ρ* determines the convexity of the utility function (Figure 1). Often times this parameter is referred to capturing “outcome” or “reward sensitivity” of an individual (Kellen et al., 2016). When considering risky choices rewards are not certain; they occur probabilistically. The subjective utility of a probabilistic reward is then simply described as:

(2) $$\mathrm{EU}=p^{*}\,V^{\rho },$$

where *p* denotes the probability of the reward. Note that in more elaborate models, such as cumulative prospect theory, the probability itself is also transformed to a subjective probability weight (Tversky and Kahneman, 1992). Although this would allow for even more detailed insights in developmental differences in risky behavior (Engelmann et al., 2012), we do not further consider subjective probability here, as it would exponentially increase our model space and thus not serveour purpose.

When individuals are repeatedly presented with the same choice options, their decisions will most likely differ from oneanother. Consequently, we need to account for this probabilistic nature of choice in a model of behavior. To achieve this, a model for choosing between two rewards feeds the difference between reward utilities into a sigmoid function, through which we obtain an estimate of the *probability* that a decision maker chooses one option over another

(3) pC⁢h⁢o⁢o⁢s⁢e⁢R⁢i⁢s⁢k=11+e-(EUrisk-EUsafe)⁢τ*.

Here, τ accounts for individual differences in choice sensitivity. The smaller τ the less sensitive the decision maker is to the expected utility differences (and the more random the choice pattern appears). We now turn to examine how different models of social decision making can be represented within this framework.

### Modeling Social Influence

In our earlier example, we used the subjective value of objective monetary amounts as the key variable for decision making, but there is ample evidence that people also attribute utility to social outcomes such as fairness (Fehr and Schmidt, 1999) and social status (van den Bos, 2009). Furthermore, there is evidence that humans integrate value information from social and non-social sources into a common currency when making a choice (see Ruff and Fehr, 2014, for a review). Consequently, the expected utility framework can be extended to include social rewards and represent social behavior.

#### Social Sensitivity

**FIGURE 2:**
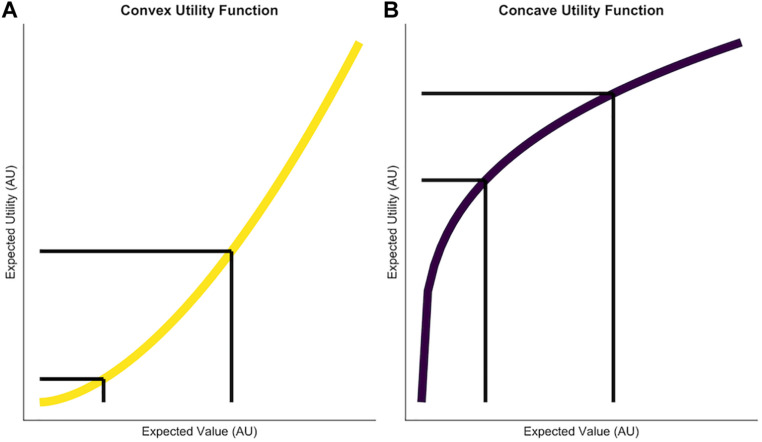
Two utility functions which are used to model reward sensitivity and risk-taking. The *x* axis depicts the expected value of potential choice options. The *y* axis shows the subjective utility of these expected values given different reward sensitivity parameters. **(A)** A convex utility function generated by *ρ* = 1.7. The difference between reward magnitudes is subjectively amplified, which makes it more attractive for the individual to take risks in order to obtain higher rewards. **(B)** A concave utility function generated by *ρ* = 0.3. Risk aversion occurs here because potential rewards are compressed, therefore more similar to each other and in turn it will be less attractive to take a risk in order to obtain the higher reward. The black lines illustrate that while the difference in expected values is equal in both graphs, the difference in subjective utility of these options is smaller in the right figure. Axis ticks and labels are not shown to, to emphasize the relative, not the absolute difference as exponential functions scale very differently.

Social rewards, such as belonging or expected status gains, can add to the expected utility associated with a non-social decision, because the prospects of social and non-social rewards are combined by the brain when making a choice (Ruff and Fehr, 2014).Within expected utility theory, the changed valuation of an option due to the presence of social information can be expressed as a single parameter that shifts subjective utility. For example, if we consider a typical experiment where there are two options, a relatively safe option and a risky option (defined by outcome variance differences). A social signal, for instance seeing that a peer chose the risky (safe) option, contributes to the utility of the risky (safe)option, while the expected value of the choice option and reward sensitivity remains the same (Chung et al., 2015). This can be implemented with a single additional parameter:

(4) $${\mathrm{EU}}_{\mathrm{Social}}=p^{*}\,V^{\rho }+\psi ,$$

where *ψ* corresponds to the impact of social information on risky and safe choice options. We call this model “symmetric social influence model.” The larger *ψ* the more likely the participant is to move into the direction of the social information (see Figure 2A).

It is likely that social information has asymmetric effects on behavior depending on whether social information favorsrisk aversion or risk seeking. For instance, Braams et al. (2019) showed that risky advice had less impact than safe advice. This can be captured by adding two independent parameters to the utility function that vary depending on whether social information favors safe or risky choices (see Figure 2B).

(5) $${\mathrm{EU}}_{{\mathrm{Social}}_{\mathrm{Risk}}}=p^{*}\,V^{\rho }+\psi_{\mathrm{risky}}\,\forall \mathrm{Social}\,\mathrm{Signal}=\mathrm{Risky},$$

$${\mathrm{EU}}_{{\mathrm{Social}}_{\mathrm{safe}}}=p^{*}\,V^{\rho }+\psi_{\mathrm{safe}}\,\forall \mathrm{Social}\,\mathrm{Signal}=\mathrm{Safe}.$$

We call this model “asymmetric social influence model.” Note that the precise interpretation of *ψ* depends on the specifics of the experiment. In an experiment where the participant is observed it could represent the expected value of gaining status by taking more risks. In an experiment where the participant observes, social information can reduce the participants uncertainty about what to choose, which will then be reflected in *ψ* and in yet another experiment, Ψ can represent the value attributed to conforming to the behavior of others (e.g., status vs. belonging motivation). In addition, such a framework offers insight in how different aspects of the outcomes are weighted (e.g., money vs. social gains).

#### Reward Sensitivity

Developmental theories on social impact that focus on imbalance suggest that in a social context, rewards are valued more by adolescents because the socially induced arousal triggers reward-processing brain regions (Chein et al., 2011). Reward sensitivity is a basic feature of expected utility models; it is governed by parameter *ρ* (see Equation 1). This parameter has already been used to characterize individual and developmental differences in risk attitudes (e.g., Blankenstein et al., 2016; van den Bos and Hertwig, 2017). To capture changes in reward sensitivity due to social facilitation one can add a parameter *ω* to the “reward sensitivity” part of the utility function:

(6) $${\mathrm{EU}}_{\mathrm{social}}=p^{*}\,V^{\left(\rho +\omega \right)}\,|\omega \in \mathbb{R}:\omega >\,0.$$

The larger *ω* the more risk seeking an individual becomes (see Figures 1, 2C). This equation will henceforth be called “reward sensitivity model.” In our reading of verbal reward sensitivity models, *ω* will never be smaller than 0 given that it is the expectation that is there is an increase, not a decrease, in risky behavior due to arousal.

#### Distraction

Other work emphasizes that arousal in social situations creates distracting goal conflicts, especially for adolescents (Dumontheil, 2016; Dumontheil et al., 2016; Botdorf et al., 2017; Breiner et al., 2018). For choices that are value- or preference-based, it is hard to judge whether a decision results from distraction or inattentiveness; there is no objectively correct benchmark to evaluate correct and incorrect responses. However, formal modeling provides the means of unmasking choice stochasticity unique to social contexts that could otherwise be falsely interpreted as an increase or decrease in risk taking. Distraction or inattentiveness would lead to an increase in choices that are less determined by expected value. In decision models this kind of behavior is often captured by a “trembling hand” choice rule (Loomes et al., 2002). This rule modifies the choice function by adding a fixed probability that the individual does not use expected utility to guide their choice, but rather chooses randomly. To capture this increase in distraction we can estimate how this probability of choosing randomly increases in the social context:

(7) $${\text{p}}_{\mathrm{Choose}\,\mathrm{Risk}\,\mathrm{Social}}=\left(1-\zeta \right)\,\frac{1}{1+{\text{e}}^{-{\left({\text{EU}}_{\text{risk}}-{\text{EU}}_{\text{safe}}\right)}^{*}\,\text{τ}}}$$

$$+\frac{\zeta }{2}|\zeta \in \mathbb{R}:0<\zeta <1,$$

where a larger ζ indicates more random behavior. We will refer to this equation as the “social distraction” model. Note that more random behavior means an increase in risk taking when one would normally show risk averse behavior, and vice versa (see Figure 2D).

#### Model Predictions

**FIGURE 3:**
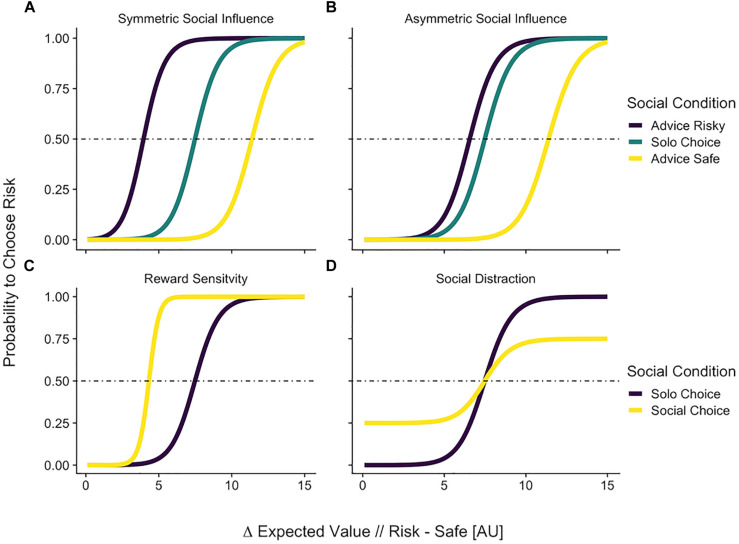
Predictions of the formal social models. The *x*-axis shows the difference in expected value of two choice options. The *y* axis shows the probability that a decision maker would choose the risky option. The horizontal line indicates the chance level for binary choice. The choice probabilities shift as a function of social information. Top panel: Predictions of models that take the content of social information into consideration. **(A)** Symmetric social influence in Eq. 4. Risky and safe social information impact choice equally. **(B)** Asymmetric social influence of Eq. 5. In this model, risky and safe social information can impact choice differentially. Bottom panel: Predictions of models that do not take the content of social information into consideration. **(C)** A reward sensitive decision maker (Eq. 6) would always be more likely to choose risky in a social context. **(D)** A distracted individual’s choices (Eq. 7) would be closer to chance in a social condition, even when the differences in expected values are extreme.

These formalizations of the different psychological processes involved in social influence make distinguishable predictions (Figure 3). Only the social influence models clearly predict that behavior will shift in a way that is dependent on the social information content (e.g., other advice is safe or risky), or the beliefs of the subject (e.g., believe the norm is safe or risky). In contrast, for reward sensitivity or distraction models, the social context has a unidirectional main effect on behavior. The fact that the models can generate different patterns of behavior is in itself no proof that these models are actually distinguishable and suitable for model comparison. For this we need to run simulations as well as model and parameter recovery analyses in the context of specific experimental settings (Palminteri et al., 2017), which we will do below.

## Methods

### Simulation Study and Model Quality

To assess the quality of our formal models, we first simulated decisions on a set of risky gambles with varying expected value, based on all outlined models. We explicitly included the standard expected utility model without any social parameters (Eq. 2), to control for the possibility that expected utility is itself flexible enough to describe a wide range of choices. We simulated decisions in a classic economic paradigm that requires repeated choices between a probabilistic lottery with a high reward and a non-probabilistic small reward. Lotteries combined values 8, 20, and 50 (Arbitrary Units) with winning probabilities 0.125, 0.25, 0.375, 0.5, 0.675, and 0.75. The safe reference always had an expected value of 5. These values resemble those used in Blankenstein et al. (2016). Social information provided in the simulations consisted of the choices of one risk seeking subject in Blankenstein et al. (2016). For each social influence model, agents were divided into 12 different groups based on the distributions used to sample the parameters ψ or which represent behavior change in the face of social information in a given model (see Table 1 for details). Reward sensitivity, ρ and temperature, τ were sampled from the same distributions for all individuals, with sufficient statistics ρ∼*N*(μ = 0.4,σ = 0.3) and τ∼*N*(μ = 0.8,σ = 0.1). For each group and model, we simulated 50 individuals, resulting in a total of 5^∗^12^∗^50 simulated subjects that responded to 432 choice problems, 144 of which contained risk seeking social information, 144 risk averse social information which was generated by inverting the choices in the risk seeking condition and 144 featured no social information. To summarize, we modeled the behavior of subjects over a range of variables of risk- and social preferences and simulated how they would respond to different choice problems in the presence of social information. We then investigated to what extent we could correctly identify the underlying data generating models, by fitting all models to the responses we generated.

**TABLE 1:** Characteristics of the simulations used for model and parameter recovery.

**Model**	**Social Parameter**	**Groups**
Symmetric social information	ψ∼*N*(μ_1_,0.5)μ ∈ *G*_1_	*G*_1_ = {0.0;0.45; 0.90; 1.36; 1.81; 2.27; 2.72; 3.18; 3.63;4.19; 4.54;5.0}
Asymmetric social information	ψ_*risk*∼_*N*(μ_2_,0.5)μ_2_ ∈ *G*_2_ψ_*safe*∼_*N*(μ_3_,0.5)μ_3_ ∈ *G*_2_	*G*_2_ = {0.0;0.45; 0.90; 1.36; 1.81; 2.27; 2.72; 3.18; 3.63;4.19; 4.54;5.0}
Reward sensitivity	ω∼*N*(μ_4_,0.5)μ_4_ ∈ *G*_3_	*G*_3_ = {0.0;0.45; 0.90; 1.36; 1.81; 2.27; 2.72; 3.18; 3.63;4.19; 4.54;5.0}
Social distraction	ζ∼*N*(μ_5_,0.1)μ_5_ ∈ *G*_4_	*G*_4_ = {0.0, 0.09, 0.18, 0.27, 0.36,0.45, 0.54, 0.63,0.72,0.81,0.90, 1.00}

*For every model (left column), the social parameter (middle column) was sampled 12 times without replacement from the tuple in the right column, resulting in 12 unique groups per model that differ in their susceptibility to social influence.*

### Model and Parameter Recovery

We evaluated all models with regard to their fit to the data we had previously generated. This enabled us to check whether our analysis was suitable to correctly identify the data generating model. That is, if successful, model fitting and comparison would indicate that the best fitting model was the one we used to generate the data. Only then can one confidently use these models to test specific hypotheses (Palminteri et al., 2017). We judged the fit of all five models given the simulated data by consulting the deviance information criterion (DIC). Lower DIC values indicate better model fit. The rule of thumb cautiously introduced by Spiegelhalter et al. (2002) is to treat DIC values higher than 3–7 relative to a better fitting reference model to be considerably less supported by the data.

It is possible that different parameter values of a model result in the same pattern of behavior. To rule out the possibility that our models are “sloppy” in that respect, we correlated the generative parameter values with the mean of the posterior parameter distribution which we obtained by inverting the generative model on itself. A high correlation between the simulation parameters and the parameter estimates obtained from inverting the data generating model on itself is indicative that we can approximate the “true” parameter values well, when inverting the model on human choices.

### Fitting Hierarchical Bayesian Models of Social Influence

We formulated the models introduced above in a hierarchical Bayesian way. This was advantageous because individual parameters could be pulled from group specific hyper distributions, which made us more sensitive to identify differences between groups and reduced outliers that often occur using frequentist fitting procedures. In our case, we drew parameters form hyper distributions for each group separately, specifying the same prior for each age group (graphical model and priors in the supplement). Non-centered parametrization was used to effectively sample subject-level parameters from the hyper distributions (Betancourt and Girolami, 2013). We obtained posterior parameter distributions using the No-U-Turn-Sampler implemented in Stan (Carpenter et al., 2017). For each model, we used 6,000 iterations of four parallel chains each and no thinning. The first 1,000 samples were discarded as warmup.

## Results

**FIGURE 4:**
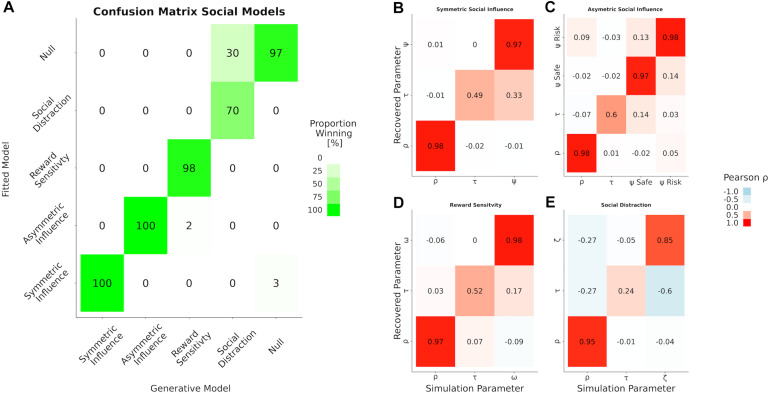
Results of the model and parameter recovery. **(A)** Confusion matrix for model recovery. Each cell depicts the frequency with which each model is best predictive for data generated under itself (columns) and inverted by itself and all other models (rows). Elements that diverge from the diagonal are evidence that one model is at danger to be “confused” with another one. The four panels on the right show the parameter recovery as correlation between the parameters used for simulation (columns) and those obtained by inverting the model (rows) for our different social influence models. **(B)** The symmetric social influence model, **(C)** the asymmetric social influence model, **(D)** the reward sensitivity model, and **(E)** social distraction model.

The results of our analyses indicate a good model and parameter recovery (see Figure 4). However, not all models performed equally well. While all Markov chains converged as indicated by the Gelman Rubin statistic (Gelman and Rubin, 1992) and most of the parameters could be recovered reliably, the trembling hand error term ζ of the social distraction model was not recovered very well (0.24 on the diagonal). Additionally, ζ was negatively correlated with the temperature parameter of the choice function (*r* = −0.6). Functional attribution to either of these parameters should be made with caution in case the social distraction model is best descriptive of the data.

## Applying the Models Synthesizes Seemingly Divergent Experimental Results

Having established that our proposed formal models and their parameters were recoverable, we applied the formal social influence, reward sensitivity and distraction models to data of two published studies, in order to quantify to what extent the studies support either model. Both studies investigated social influence when adolescents observed social information as they chose between different monetary lotteries (Blankenstein et al., 2016; Braams et al., 2019). Using our formalized versions of models on social influence in adolescent risk taking, we investigated how well either study supported social information, reward sensitivity or distraction models. Both studies provided the participants with safety- and risk-promoting social information. The studies investigated how explicit information about another person’s choices changed risk-taking behavior in monetary lotteries and how this change in risk-taking was related to the participants’ developmental stage. The combined age range of both studies was 10–26. The first study focused on adolescent reward sensitivity and reported that social impact on risk-taking decreases with age (Blankenstein et al., 2016). The other focused more on adolescent social motivation and found that social impact for safe behavior was strongest in adolescence (Braams et al., 2019). Notably, these two studies used very similar paradigms but report results in line with different verbal models of adolescent risk-taking in social contexts. Below we will re-analyze both studies and show that formal model comparison can synthesize these seemingly divergent explanations. Our re-analysis was restricted to these two studies because these studies are so similar which made a straightforward showcase for the benefits of formal modeling.

### Analysis

In both datasets, we compared the formal models via DIC. The experimental paradigms included risky choices where the probability was known, and ambiguous choices where the exact probabilities where not known. For sake of simplicity, we have currently ignored the ambiguous trial types in the main manuscript. However, we believe that the discussion of risk, ambiguity or even experience-based choice in relation to adolescent risk-taking is very important, but beyond the scope of the present paper [but see Rosenbaum et al. (2018) for review]. Thus, in this articles’ Supplementary Material we report how we adjusted the formal models to include an ambiguity attitude parameter (Tymula et al., 2012; van den Bos and Hertwig, 2017) and repeated all analyses with expected utility and ambiguity models. The main results of the model comparison remained the same (see Supplementary  Material).

For inference on age trends in the best fitting models’ parameters, we used Bayesian general linear models, implemented in the rstanarm package (Stan Development Team, 2018) utilizing rstanarm’s default priors. The age predictor was binned to represent pre- (age < 13), early- (age 13–16), late- (age 16–19), and post-adolescent (age > 19) groups. To test linear and quadratic age trends we constructed orthogonal regressors using R’s poly function. We subsequently inverted the quadratic age predictor, so that the beta estimates were more positive, when its contribution to the dependent variable increases. For each regression, we ran 3 chains with 30,000 samples each and set a warmup of 1,000 samples. Convergence of the chains was inspected by consulting stan’s implementation of the Gelman Rubin statistic (Gelman and Rubin, 1992). Generally, we report the mean of the posterior and the two-sided 95% credible intervals (CI) around each mean. We treat the contribution of a predictor as negligible if the credible interval of the regression weights includes a zero.

### Experiment 1: Reanalysis of Blankenstein et al. (2016)

Blankenstein et al. (2016) tested *n* = 157 participants aged 10–26. In this study, participants were asked to choose between a risky gamble and a safe option on 216 trials. In order to investigate susceptibility to peer influence, Blankenstein et al. (2016) programmed a virtual agent very prone to risk-taking and showed its choices to the participants prior to their choice in half of the trials. Note that this agent was very risk-taking on average, but sometimes chose the safe option as well. The probabilities and values associated with the gambles were presented as wheels of fortune (Ernst et al., 2004) and were the same as the ones we used for our simulations. A full combination of gain values and probabilities resulted in 24 unique trials. The authors reported an overall increase in risky choices in the social condition; the increase was most pronounced in youngest participants and linearly diminished with age. The original analysis was motivated by the reward sensitivity model. The study design and their model-based analysis focused on a change in risk attitude as measured by the reward sensitivity parameter, ρ, which we introduced earlier. However, their reported result is, in principle, consistent with all three verbal models of risk-taking under consideration in this paper. Our reanalysis goes beyond the original analysis as we specifically designed formal models to compare competing models about the nature of social influence during adolescence within the same task.

#### Results

**FIGURE 5:**
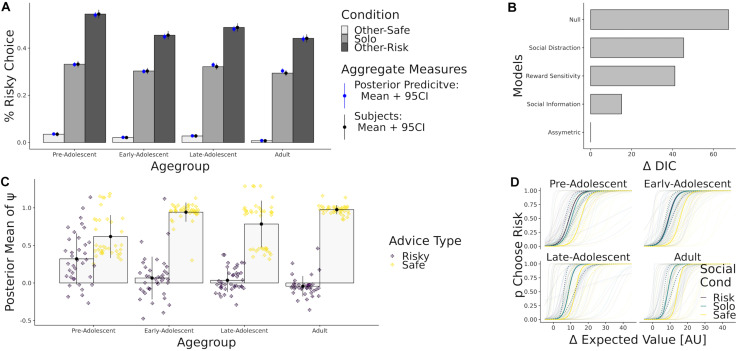
This panel shows the results of our model comparison procedure. **(A)** Percent risky choice in Blankenstein et al. (2016), by age group and conditions. Black error bars represent the bootstrapped 95% confidence interval. Next to the mean and CI of the subjects choices (black), we show simulations under the full posterior from the winning model’ parameter estimates (blue). **(B)** Difference in DIC fit indices for the whole modelspace, using the winning model as a reference. **(C)** Posterior Parameter Estimates of ψ _*risk*_ (purple) and ψ _*safe*_ (yellow), binned by age group. **(D)** Predicted probability to choose the risky option given the difference in expected value of the gambles. Colored solid lines correspond to model predictions obtained by computing the mean of subject-level parameters in each age group. Colored dashed lines denote upper and lower confidence bounds obtained by computing the standard error of the posterior mean. Transparent lines refer to subject-level predictions.

Model comparison via DIC identified the asymmetric social influence model as best fitting (Figure 5B). Reward sensitivity and distraction models performed considerably worse in comparison. The best fitting model replicated the behavior of participants with great accuracy (Figure 5A). All age groups made more risky decisions when social information was risky, and made more safe choices when social information was safe. Strikingly, and contrary to our expectations given the original article, *all* participants showed a greater social influence when being confronted with safety-promoting social information (Figure 5C). As a next step, we performed Bayesian generalized linear regressions using age and quadratic age as predictor of the social model parameter estimates. We ran separate regression analysis on ψ _*risk*_ and ψ _*safe*__,_ treating them as separate dependent variables. We found that the older the participants, the less they took risky advice [β_*AgeLin*_ = −1.5, CI = (−1.9, −1.2)]. Older participants additionally took safe advice more often [β_*AgeLin*_ = 1.4, CI = (0.9, 2.0)] as compared to younger participants. We also found adolescent decrease in taking risky advice as indicated by a negative quadratic contribution of age for following risky [β_*AgeQuad*_ = −0.6 CI = (−0.9, −0.2)] but no adolescent effects on taking safe advice [β_*AgeQuad*_ = 0.5 CI = (−0.0, 1.1)]. In sum, participants of all ages were influenced by both safe and risky social information. In agreement with the original author’s conclusions, we found that the impact of risky social information was strongest in youngest participants. Crucially however, safe social information had an even stronger impact than risky social information in all age groups, a conclusion which was not noted in original analyses.

### Experiment 2: Reanalysis of Braams et al. (2019)

In this experiment *n* = 99 participants aged 12–22 chose between risky and safe gambles on 300 trials. Similar to Blankenstein et al. (2016), the authors were interested in the impact of social information on risky choice across development and presented subjects with computer generated decisions that participants believed were choices from other participants of the study. Other than in Blankenstein et al. (2016), risky and safe options were both gambles with equal probabilities: there was no sure option to choose from. In both gambles, it was either possible to win a low or a high reward. Risky gambles could result in either very low or very high rewards. For the risky options, the difference between the high and low rewards varied from $3.63 to $5.51. For safer options, there was less to lose as the difference was between $0.06 and $1. The probability of winning the high reward varied with a step size of 10% from 40% up to 90%. The lotteries were presented as colored bars, with color proportions indicating the winning probability. The authors concluded that participants followed risky and safe choices of peers and that adolescents use safe more than risky social information. Such a result speaks for social motivation models. However, as seen above: drawing conclusions about mechanisms is hard without a formal model comparison. In order to be able to apply formal model comparison here, the models were adapted to reflect the conceptualization of risk as the variability in outcomes (Weber et al., 2004) used in Braams et al. (2019). Hence, the utility of one choice option in this re-analysis is described as:

(8) $$E\,U=p*V_{h\,i\,g\,h}^{\rho }+\left(1-p\right)*V_{l\,o\,w}^{\rho }$$

while the social extensions to this model remained the same.

#### Results

**FIGURE 6:**
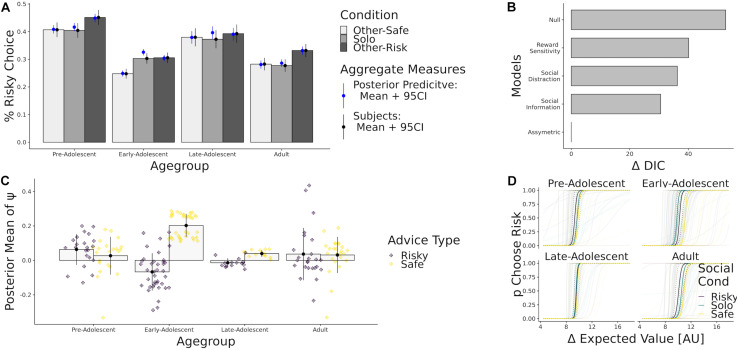
**(A)** Percent risky choice and model simulations (blue) by age group and conditions. **(B)** Difference DIC fit indices for the considered model space, using the winning model as a reference. **(C)** Mean of the posterior parameter estimates of ψ _*risk*_ (purple) and ψ _*safe*_ (yellow), binned by age group. **(D)** Predicted probability to choose the risky option given the difference in expected value of the gambles.

Model comparison via DIC again indicated, as in Blankenstein et al. (2016), that the asymmetric social influence model fit the overall behavior best (Figure 6B). Again, reward sensitivity and distraction models had considerably worse fit than the models which assume that social impact depends on the content of social information. Simulating data under the obtained posterior distributions again revealed that our models could predict the participant choices well (Figure 6A).

As before, most participants put higher weight on safety-promoting social information than on risky social information (Figure 6C). To judge the statistical relevance of this pattern, we performed Bayesian generalized linear regressions, again using age and quadratic age as predictors while treating ψ _*risk*_ and ψ _*safe*_ as separate dependent variables. We found that linear age was not a good predictor for using risky [β_*AgeLin*_ = 0.0, CI = (−0.2, 0.3)] nor safe advice [β_*AgeLin*_ = −0.2 CI = (−0.4, 0.0)]. However, quadratic age trends were substantial for both risky [β_*AgeQuad*_ = −0.5 CI = (−0.7, −0.3)] and safe advice [β_*AgeQuad*_ = 0.6 CI = (0.4, 0.8)], implying that adolescents used risky social information less and safe social information more to guide their choice. In sum we find that safe social information has a greater impact on choice than risky information, especially so during adolescence. Again, model comparison provides evidence that all age groups differentially assign weight to risky and safe social information.

## Discussion

It is a widespread assumption that adolescents take risks more frequently and are more sensitive to social information than members of other age groups. Why this is the case, and in which situations this occurs remain open questions despite extensive theory development and empirical research. Several verbal models of adolescent decision-making have identified elements that may play a role in increased risk-seeking behavior. Some point to high social motivation as the principle driving force of adolescent decision-making. Others emphasize reward sensitivity or increased arousal in social situations; yet others have focused more on diminished cognitive control and increased distraction in social contexts. Most of the current experimental evidence is consistent with more than one of these explanations, resulting in a handful of plausible verbal models that explain social influence in adolescent risk-taking well. Further progress requires the systematic testing of models against each other, within different social contexts (Pfeifer and Allen, 2016; van den Bos and Eppinger, 2016). With this goal in mind, we translated verbal models of adolescent social decision-making into formal models (c.f. Figure 1), which make distinguishable quantitative predictions (c.f. Figure 3). Using simulations and Bayesian model inversion, we first demonstrated that these models can be recovered and thus can be tested against each other using a single experimental setting (c.f. Figure 4). We then reanalyzed two published studies investigating the development of social influence, and showed how the model-based approach can synthesize the results of two studies on social observation (c.f. Figures 5, 6). Here we will discuss the implications of our findings regarding the re-analyzes and, more importantly, the general applications and limitations of the modeling approach. Additionally, we provide specific suggestions for research on social influence in adolescence.

### Adolescents Are Influenced More by Safe Social Information Than Risky Social Information

Even though the reanalyzed studies (Blankenstein et al., 2016; Braams et al., 2019) share a similar paradigm, they are different in terms of stimuli (wheels of fortune vs. bars), reward magnitudes (high vs. low), choices (risky/safe vs. low/high risk), and the source of social information (peer vs. peer/computer/non-peer). This resulted in considerable differences in the aggregate behavior of the subjects (Figures 5A, 6A). However, applying our models yielded similarities between the two studies which were not easily gleaned from the original articles. First, we showed that in both experiments, participants of all ages took safety and risk promoting social information into account. This is consistent with the original interpretation of Braams et al. (2019), but not clear from the original analysis of Blankenstein et al. (2016). Second, model comparison and the parameter estimates of both re-analyses indicated that safer social information consistently weighs stronger than risky information, especially for adolescents. We can therefore conclude that, when risk aversion is valued by peers, social information can induce safety-promoting behaviors in adolescents. This is worth emphasizing because assuming that adolescent decision-making is maladaptive or flawed is unhelpful in designing social interventions. Restrictive public interventions solely based on that notion have been at best only mildly successful in making adolescents “better” decision-makers in the past (Albert and Steinberg, 2011; Rosenbaum et al., 2018). Mobilizing the finding that social information can favorize safe decision-making could lead to better interventions and perhaps reduce dangerous real-world risk-taking. Taken together, our results confirm a positive outlook on adolescent decision-making and add further evidence that adolescent social motivation can be used for the good (Perkins et al., 2011; Liu et al., 2017; Telzer et al., 2018; van Hoorn et al., 2018). However, it is important to note that our conclusions are limited to paradigms where social information is passively observed; it may well be very different when applied to data of experiments where the participant was observed by others.

### Computational Modeling Can Inform Experimental Design

In principle, formal models make it possible to quantify social impact in various contexts and increase the specificity of a given hypothesis, but they are no panacea. Models require well-designed experiments: the conclusions that can be drawn from model parameter estimates and model comparison depend on the experimental paradigm. For example, as previously noted, experiments where the participant is observed could lead both to unspecific arousal and also to specific social messaging by the participant (e.g., signaling they are a risk-taker to gain status). Both phenomena can lead to an increase in risk-taking, and this behavior can be consistent with all of our formal models. In order to be able to draw informative conclusions about social mechanisms, experiments need control conditions where adolescents can achieve social status by demonstrating safe behavior. Similarly, an experiment where participants only observe the risky decisions of others is by design unable to generate support for a social sensitivity model, and would likely furnish behavioral data consistent with all models, verbal or formal. The relationship between formal model parameters and experimental elements additionally allows for an unambiguous specification of the conditions needed to distinguish between models, fostering better experimental design. Model simulations can be used *a priori* to show whether the specific implementation of a proposed experiment can distinguish between models^1^. In other terms, although formal models cannot compensate for poorly designed experiments, they significantly contribute to the development of experimental designs that generate testable hypotheses.

### Computational Models Can Help Interpret Neuroimaging Results

All theories about the nature of adolescent decision-making are supported by neurodevelopmental research using techniques like (f)MRI. However, the often-used practice of reverse inference from observed neural activity about the engagement, or the absence of a specific cognitive process is problematic (Poldrack, 2006, 2011). Formal models are helpful in order to overcome some logical fallacies associated with reverse inference (Poldrack, 2011). When using formal modeling, the engagement of cognitive processes is quantified by comparing plausible process models which are subsequently fitted to observed behaviors. In the example of expected utility models, used throughout this article, formal modeling provides insight into the otherwise hidden process of subjective utility computation. Crucially, model comparison happens *before* regressing the winning models’ parameter estimates to measured neuronal activity. Inference can thus be made more rigorously, avoiding logical aberrations such as assuming that activity in the mPFC solely equates social motivation, whereas this activity could also reflect reward sensitivity. Additionally, the model-based approach helps the understanding of developmental processes (van den Bos et al., 2017). In summation, computational modeling is useful to attenuate some issues associated with reverse inference and can lead to more detailed, process-based insights about cognitive development.

### Limitations and Future Directions

Naturally the current article is not free of caveats, some of which we will discuss in the following section. Most strikingly, our results only apply to two paradigms in which people observe behavior, and thus we cannot conclude that this pattern generalizes to behavior where participants are being observed. Real-life decisions are additionally more complex than the decisions in the binary choice tasks we have highlighted here. In the real world it is rare to be presented with accurate information about outcomes and probabilities associated with choices; there are usually multiple sources of uncertainty (Bach and Dolan, 2012). Although beyond the scope of the current article, there are several computational frameworks that aim at understanding behavior under different types of uncertainty. This can take different forms such as ambiguity extensions of expected utility (Tymula et al., 2012; van den Bos and Hertwig, 2017) or Bayesian decision frameworks, which assume that social influence is stronger when individuals are more uncertain (Toelch and Dolan, 2015). There has been much attention on the distinction between risk and ambiguity in the literature; both datasets reanalyzed here also originally tested age trends in attitudes toward ambiguity. We did not focus on the ambiguous trials in the main article, as our focus was on formalizing verbal models. However, there is reason to expect that ambiguity increases social influence (Toelch and Dolan, 2015) which is why we repeated the same analysis using ambiguity extensions and classical expected utility models (see Supplementary Material). This did not affect the conclusions of our model comparison. We encourage further studies that investigate if the social parameters of the models differ between risky and uncertain or ambiguous choices. Of additional note is that in real life there is not only uncertainty about what to choose, but real-life knowledge of probabilities and outcomes is acquired dynamically through experience (Hertwig and Erev, 2009). Learning in dynamic environments can be modeled within the reinforcement learning framework (Dayan and Niv, 2008), which can be adapted similarly to the models we proposed here in order to comprehend the development of social influence in experience-based tasks (Behrens et al., 2008; Biele et al., 2011; Diaconescu et al., 2014; Bolenz et al., 2017; Rodriguez Buritica et al., 2019).

As briefly mentioned affect is another important modulator of adolescent risk-taking. In affectively arousing (i.e., “hot”) contexts, adolescents make risky decisions more often than in less arousing (i.e., “cold”) contexts (Figner et al., 2009; Defoe et al., 2015; Laube and van den Bos, 2016; Rosenbaum et al., 2018). In fact, social facilitation theory as well as reward sensitivity and distraction models all imply that social behavior is influenced by arousal, which itself is often understood as affectively hot. Therefore, research on social influence needs to closely examine the interaction between affect and social processing. “Cold” social situations might be where the participant is merely observing others and “hot” situations might be those where the participant is being observed or interacts with others. However, we believe that a one-to-one mapping between social and affective contexts seems overly simplistic. In the future, it will be interesting to see how different processes like reward sensitivity, social motivation or distraction have different weights in different affective contexts and how strong affect mediates behavior change. From our current understanding of the literature, “hot” contexts might be best described with reward sensitivity or distraction models whereas behavior in “cold” contexts might be better described by models emphasizing social motivation. Careful experimental design in combination with formal models may delineate the importance of each process in explaining developmental changes in peer influence.

## Summary and Conclusion

Adolescents are often thought to be excessive risk-takers, especially in social contexts. Since adolescents’ risky decisions constitute a major health hazard and can have long term consequences, several attempts have been made to understand the determinants of adolescent social risk-taking. Plausible verbal models of social influence in adolescent risk-taking have been formulated, but it is difficult to identify which of the proposed processes determine adolescent behavior in a particular social situation. We argue that this is because verbal models make unspecific predictions: a broad range of observations is consistent with one, or even several, verbal models. Here we make a first attempt to specify models of social influence in adolescent risk-taking by connecting the developmental literature to theories of social psychology and representing them as simple formal models. Reanalyzing two published studies on social influence in risky choice yields that adolescents, like adults, are sensitive to the quality of social information and carefully integrate it into private decisions. In both studies, safe social information had a stronger influence than risky information on adolescents’ decisions. These results add further evidence that adolescent social sensitivity can result in safe, health promoting behavior. Investigating if and how this pattern generalizes to other contexts for instance when adolescents are being observed, will be most insightful. We hope this article encourages further work on isolating the building blocks of developmental models, through harnessing the specificity of formal modeling and model comparison.

## Author’s Note

SC is a pre-doctoral fellow of the International Max Planck Research School on Computational Methods in Psychiatry and Aging Research (IMPRS COMP2PSYCH). The participating institutions are the Max Planck Institute for Human Development, Berlin, Germany and the University College London, London, United Kingdom. For more information, see https://www.mps-ucl-centre.mpg.de/en/comp2psych.

## Data Availability

The data and code to reproduce all simulations, figures, and model fitting in this study can be found on github at: https://github.com/NomisCiri/Social_Adolescence_Public.

## Ethics Statement

For the dataset from the study in Blankenstein et al. (2016), written informed consent was provided by the participants themselves or by a parent in the case of minors. Recruitment, written informed consent, and procedures were approved by the local ethics committee in Leiden. The study reported in Braams et al. (2019) was approved by the Committee on the Use of Human Subjects at Harvard University.

## Author Contributions

SC and WB designed the research and wrote the manuscript. SC planned, performed, and visualized the data analysis.

## Conflict of Interest Statement

The authors declare that the research was conducted in the absence of any commercial or financial relationships that could be construed as a potential conflict of interest.

## Footnotes

1 https://github.com/NomisCiri/Social_Adolescence_Public/tree/master/A_Simulations
