# Corrigendum: Social Influence in Adolescent Decision-Making: A Formal Framework

**DOI:** 10.3389/fpsyg.2020.598347

**Published:** 2020-12-03

**Authors:** Simon Ciranka, Wouter van den Bos

**Affiliations:** ^1^Center for Adaptive Rationality, Max Planck Institute for Human Development, Berlin, Germany; ^2^Max Planck UCL Center for Computational Psychiatry and Ageing Research, Berlin, Germany; ^3^Department of Developmental Psychology, University of Amsterdam, Amsterdam, Netherlands

**Keywords:** adolescence, decision-making, social influence, risk-taking, expected utility, computational modeling, hierarchical bayes

In the original article, we neglected to include the following funding information. WB was supported by Open Research Area (ID 176), the Jacobs Foundation, the European Research Council (ERC-2018-StG-803338) and the Netherlands Organization for Scientific Research (NWO-VIDI016.Vidi.185.068).

Additionally, in the original article, there was an error. The equations in the original article had brackets where they did not belong, which may be confusing to researchers who attempt to reproduce the article. The analysis code and GitHub repository contained the correct implementation, therefore the work in the article is not affected by these mistakes.

Corrections have been made to all paragraphs in the following sections: *Formal Models of Social Influence, Expected Utility, Modeling Social Influence, Social Motivation* (now renamed as *Social Sensitivity*)*, Reward Sensitivity, and Distraction*.

The corrected paragraphs are included below:

## Formal Models of Social Influence

Here we demonstrate how the three verbal models about adolescent socioemotional development which we introduced earlier can be formalized as variations of expected utility models. We then show that model comparison can be used to infer underlying social mechanisms. The rationale behind formal modeling of cognition is that in order to identify if behavior is consistent with a proposed cognitive process, we need to formulate algorithms that represent the process mathematically. Comparing the behavior of the algorithms with actual behavior observed in participants can subsequently be used to quantify support for the hypothesis which is represented by the algorithm. In this section, we aim to translate verbal models of adolescent development into formal ones. However, current models often lack the details required in order to be directly translated into formal models. To formalize the models, we have therefore made several assumptions rooted in expected utility theory. The model space that we present here is not exhaustive. Nevertheless, the current framework illustrates how formal modeling can be used in developmental science, and provides a strong starting point for developing more elaborate models. More importantly, it enables precise discussions on which models are favored by existing experimental data. To formalize models of adolescent decision making First, we address how risk seeking behavior is understood within the expected utility framework in order to familiarize the reader with its' assumptions. Then we extend these models with parameters that can be read as social sensitivity, reward sensitivity, and distraction. This finally enables us to test models of adolescent development against one another, even within the same experiment.

### Expected Utility

The first assumption of expected utility theory is that people have a subjective experience of objective rewards. For instance, the first dollar someone ever earns is worth more to them than the hundredth. The change in wealth from nothing to $1 feels different from the change in wealth from $99 to $100. This transformation of objectively equal values ($1 in both cases) into a subjective utility is often modeled by a power function borrowed from psychophysics (Helmholtz, 1896), where it is used to describe the non-linear relationship between subjective psychological experience of a stimulus intensity and the objective physical intensity of the stimulus:

(1) $$\begin{array}{l} \text{U}={\text{V}}^{\text{ρ}}, \end{array}$$

Where *V* denotes the objective value of a reward and ρ determines the convexity of the utility function (**Figure 1**). Often times this parameter is referred to capturing “outcome” or “reward sensitivity” of an individual (Kellen et al., 2016). When considering risky choices rewards are not certain; they occur probabilistically. The subjective utility of a probabilistic reward is then simply described as:

(2) $$\begin{array}{l} \text{EU }=\;{\text{p}}^{*}{\text{V}}^{\text{ρ}}, \end{array}$$

where *p* denotes the probability of the reward. Note that in more elaborate models, such as cumulative prospect theory, the probability itself is also transformed to a subjective probability weight (Tversky and Kahneman, 1992). Although this would allow for even more detailed insights in developmental differences in risky behavior (Engelmann et al., 2012), we do not further consider subjective probability here, as it would exponentially increase our model space and thus not serve our purpose.

When individuals are repeatedly presented with the same choice options, their decisions will most likely differ from one another. Consequently, we need to account for this probabilistic nature of choice in a model of behavior. To achieve this, a model for choosing between two rewards feeds the difference between reward utilities into a sigmoid function, through which we obtain an estimate of the *probability* that a decision maker chooses one option over another

(3) $$\begin{array}{l} {\text{p}}_{ChooseRisk}=\frac{1}{1+{\text{e}}^{-\left(\text{E}{\text{U}}_{\text{risk}}-\text{E}{\text{U}}_{\text{safe}}\right)*\text{τ}}}\;. \end{array}$$

Here, τ accounts for individual differences in choice sensitivity. The smaller τ the less sensitive the decision maker is to the expected utility differences (and the more random the choice pattern appears). We now turn to examine how different models of social decision making can be represented within this framework.

### Modeling Social Influence

In our earlier example, we used the subjective value of objective monetary amounts as the key variable for decision making, but there is ample evidence that people also attribute utility to social outcomes such as fairness (Fehr and Schmidt, 1999) and social status (van den Bos, 2009). Furthermore, there is evidence that humans integrate value information from social and non-social sources into a common currency when making a choice (see Ruff and Fehr, 2014, for a review). Consequently, the expected utility framework can be extended to include social rewards and represent social behavior.

#### Social Sensitivity

Social rewards, such as belonging or expected status gains, can add to the expected utility associated with a non-social decision, because the prospects of social and non-social rewards are combined by the brain when making a choice (Ruff and Fehr, 2014). Within expected utility theory, the changed valuation of an option due to the presence of social information can be expressed as a single parameter that shifts subjective utility. For example, if we consider a typical experiment where there are two options, a relatively safe option and a risky option (defined by outcome variance differences). A social signal, for instance seeing that a peer chose the risky (safe) option, contributes to the utility of the risky (safe)option, while the expected value of the choice option and reward sensitivity remains the same (Chung et al., 2015). This can be implemented with a single additional parameter:

(4) $$\begin{array}{l} \text{E}{\text{U}}_{\text{Social}}=\;{\text{p}}^{*}{\text{V}}^{\text{ρ}}+\text{ψ}, \end{array}$$

where ψ corresponds to the impact of social information on risky and safe choice options. We call this model “symmetric social influence model.” The larger ψ the more likely the participant is to move into the direction of the social information (see **Figure 2A**).

It is likely that social information has asymmetric effects on behavior depending on whether social information favors risk aversion or risk seeking. For instance, Braams et al. (2019) showed that risky advice had less impact than safe advice. This can be captured by adding two independent parameters to the utility function that vary depending on whether social information favors safe or risky choices (see **Figure 2B**).

(5) $$\begin{array}{l} \text{E}{\text{U}}_{\text{Socia}{\text{l}}_{\text{Risk}}}={\text{p}}^{*}{\text{V}}^{\text{ρ}}+{\text{ψ}}_{\text{risky}}\;\forall \text{ Social Signal}=\text{Risky },\\ \text{E}{\text{U}}_{\text{Socia}{\text{l}}_{\text{safe}}}={\text{p}}^{*}{\text{V}}^{\text{ρ}}+{\text{ψ}}_{\text{safe}}\;\forall \text{ Social Signal }=\text{Safe}. \end{array}$$

We call this model “asymmetric social influence model.” Note that the precise interpretation of ψ depends on the specifics of the experiment. In an experiment where the participant is observed it could represent the expected value of gaining status by taking more risks. In an experiment where the participant observes, social information can reduce the participants uncertainty about what to choose, which will then be reflected in ψ and in yet another experiment, Ψ can represent the value attributed to conforming to the behavior of others (e.g., status vs. belonging motivation). In addition, such a framework offers insight in how different aspects of the outcomes are weighted (e.g., money vs. social gains).

#### Reward Sensitivity

Developmental theories on social impact that focus on imbalance suggest that in a social context, rewards are valued more by adolescents because the socially induced arousal triggers reward-processing brain regions (Chein et al., 2011). Reward sensitivity is a basic feature of expected utility models; it is governed by parameter ρ (see Equation 1). This parameter has already been used to characterize individual and developmental differences in risk attitudes (e.g., Blankenstein et al., 2016; van den Bos and Hertwig, 2017). To capture changes in reward sensitivity due to social facilitation one can add a parameter ω to the “reward sensitivity” part of the utility function:

(6) $$\begin{array}{l} \text{E}{\text{U}}_{\text{social}}\;={\text{p}}^{*}{\text{V}}^{\left(\text{ρ}+\text{ω}\right)}\;|\text{ ω}\in \text{ℝ}:\text{ ω}>0. \end{array}$$

The larger ω the more risk seeking an individual becomes (see **Figures 1**, **2C**). This equation will henceforth be called “reward sensitivity model.” In our reading of verbal reward sensitivity models, ω will never be smaller than 0 given that it is the expectation that is there is an increase, not a decrease, in risky behavior due to arousal.

#### Distraction

Other work emphasizes that arousal in social situations creates distracting goal conflicts, especially for adolescents (Dumontheil, 2016; Dumontheil et al., 2016; Botdorf et al., 2017; Breiner et al., 2018). For choices that are value- or preference-based, it is hard to judge whether a decision results from distraction or inattentiveness; there is no objectively correct benchmark to evaluate correct and incorrect responses. However, formal modeling provides the means of unmasking choice stochasticity unique to social contexts that could otherwise be falsely interpreted as an increase or decrease in risk taking. Distraction or inattentiveness would lead to an increase in choices that are less determined by expected value. In decision models this kind of behavior is often captured by a “trembling hand” choice rule (Loomes et al., 2002). This rule modifies the choice function by adding a fixed probability that the individual does not use expected utility to guide their choice, but rather chooses randomly. To capture this increase in distraction we can estimate how this probability of choosing randomly increases in the social context:

(7) $$\begin{array}{l} {\text{p}}_{\text{ChooseRiskSocial}}=\left(1-\text{ζ}\right)\frac{1}{1+{\text{e}}^{-{\left(\text{E}{\text{U}}_{\text{risk}}-\text{E}{\text{U}}_{\text{safe}}\right)}^{*}\text{τ}}}\\ \;+\frac{\text{ζ}}{2}\;|\text{ ζ}\in \text{ℝ}:\;0<\text{ζ}<1\;, \end{array}$$

where a larger ζ indicates more random behavior. We will refer to this equation as the “social distraction” model. Note that more random behavior means an increase in risk taking when one would normally show risk averse behavior, and vice versa (see **Figure 2D**).

The authors apologize for these errors and state that they do not change the scientific conclusions of the article in any way. The original article has been updated.

