# Supplementary material for: Social Influence in Adolescent Decision-Making: A Formal Framework
Source: Front Psychol. 2019 Aug 29;10:1915. doi: 10.3389/fpsyg.2019.01915 (PMC6727856; doi:10.3389/fpsyg.2019.01915)
Supplement: Supplementary file 1 [file Table_1.DOCX]

Supplementary Material

# Hierarchical Models of social influence

The hierarchical Bayesian models we formulated had the following structure: We assumed subject level parameters to be sampled from hyper distributions that have uninformative priors. The asymmetric social influence model had two “social”parameters, one for safe and one for risky social information which are both treated exactly the same way as $\psi$ and its hyper parameters.

Supplementary Figure 1: Graphical model of all models with priors used for simulations. The equations in observed choices correspond to Eq. 4 of the main text and shall be substituted with the other models, whereas the general structure of our hierarchical model and all priors remain the same for the respective parameters.

# Model adjustments to account for ambiguity

Both studies reanalyzed by us included an additional condition which we do not explicitly analyze in the main article. Both experiments examined social influence under two kinds of uncertainty: Risk and ambiguity. Risk refers to the fact that outcomes are probabilistic. Ambiguity refers to the explicit uncertainty *about* the outcome probabilities themselves and is often referred to as “known to be missing” information. This dimension of both studies is not relevant in order to generally motivate formal modeling which was the main focus of the present article. However, it can be argued that paying credit to this distinction might change the main articles qualitative conclusion that adolescents are more influenced by safety promoting social information. The line of argument is that ambiguity increases social influence, because it increases the uncertainty about what to choose. Additionally, adolescents have previously been reported to be tolerant to ambiguity (Tymula et al., 2012) and decide overly optimistic when faced with ambiguous choices. Optimism can result in more risky choices, especially when social information favors them.

In both reanalyzed studies experiments ambiguity was achieved via covering the outcome indicators (Wheel of Fortune in Blankenstein et al., 2016 and Bars in Braams et al., 2019), in order to only disclose a 50/50 probability of winning to the participants. In both experiments, participants were told that the “real” probability was not 50/50, but the exact winning probability could be anywhere within the visual cover plus the proportion of the whole outcome indicator as seen by the participant.

# Ambiguity Extension in Blankenstein et al. 2016

In order to account for this incomplete probability information in half of the trails of both experiment we adjusted out model for ambiguous trials to include a parameter capturing ambiguity attitude, which was orthogonal to the participants reward sensitvity or “risk attitude”. missing”. In Blankenstein et al. (2016), Between 25%, 50%, 75%, and 100% (In Braams et al. (2019), 20%, 40%, 60%, 80%) of the wheel of fortune (bars) was covered in these ambiguous conditions. In our computational models ambiguity is included as a scaling factor of the 50/50 probability information as follows:

Eq.1 ${EU}_{solo} =\left( p*\beta*\frac{A}{2} \right)*\left( V \right)^{\rho} \forall Ambigous Trials$,

where $\beta$ captures the weight of the ambiguity in the choice. $\beta$ estimates larger than 0 are indicative of a pessimism meaning that the decision maker always treats ambiguous options as if ambiguity would reduce the chance of winning (Tymula et al., 2012b).

# Parameter estimates and correlations in Blankenstein et al. 2016

As parameter inference was not the focus of our main article our article and we had no hypothesis about the extent of these parameters we do not report them in the main text. However, it is notable that $\beta$ is indeed only weekly correlated with the other parameter estimates. Furthermore, age trends in all parameters reported agree with these reported in the original study.

Supplementary Figure 2: Parameter inspection for Blankenstein et al. (2016). a) Correlations within the posterior mean of the parameter estimates obtained from inverting the asymmetric social influence model on the data shown in Blankenstein et al. (2016). b)-d): Individual parameters and age trends that were not shown in the main manuscript. Error bars denote the standard deviation.

# Ambiguity Extension in Braams et al. 2019

In this study, participants did not have a safe alternative to choose from, but decided between two differently risky gambles. Therefore, each options’ utility was defined as:

Eq.1 $EU=\left( p*\beta*\frac{A}{2} \right)*{V^{\rho}}_{high}+\left( 1-\left( p*\beta*\frac{A}{2} \right) \right)*{V^{\rho}}_{low}$,

Where p denotes the probability to win the high value and ambiguity was accounted for with replacing p with the first term of the product in Eq 1.

# Parameter estimates and correlations in Braams et al. 2019

Supplementary Figure 4: Parameter inspection for Braams et al. (2019).

# Analyzing only Risky Trails in Blankenstein et al. 2016

In what follows we show the results of analyzing only risky trials in Blankenstein et al (2016). We did so because it is conceivable that ambiguity changes the amount of social influence. If this would be the case our reported results would be biased and would need to be interpreted even more cautiously.

In the ambiguity extended models of Blankenstein et al (2016), which we presented in the main article Model comparison via DIC again identified the asymmetric social influence model as best fitting (Figure 4B) for the whole dataset. Here we investigated if subsetting the data and using only risky trials changed this model comparison. It did not. Using only expected utility models and only data that was subject to risky trials is consistent to the reports in the main article: all age groups made more risky decisions when social information was risky, and made more safe choices when social information was safe. Again, all subjects were stronger influenced by safe than by risky social information (Figure 4C). Like in the main text, we then performed Bayesian generalized linear regressions using age and quadratic age as predictor of the social model parameter estimates. We ran separate regression analysis on Ψ_risk_ and Ψ_safe,_ treating them as separate dependent variables. In short: the qualitative results are exactly the same. We found that participants took risky advice less, the older they were (β_AgeLin_ = -1.5, CI = [-2,-1.1]) at the same time older participants took safe advice more (β_AgeLin_ = 1.2, CI = [0.8,1.7]). We also found adolescent specific effects as indicated by a quadratic contribution of age for following risky (β _AgeQuad_= -0.5 CI = [-1,-0.1]) but no adolescent-specific effects on taking safe advice (β _AgeQuad_= 0.5 CI = [-0.1,0.9]). In sum, participants of all ages are influenced by both safe and risky social information. In agreement with the original author’s conclusions, we found that the impact of risky social information is strongest in youngest participants.


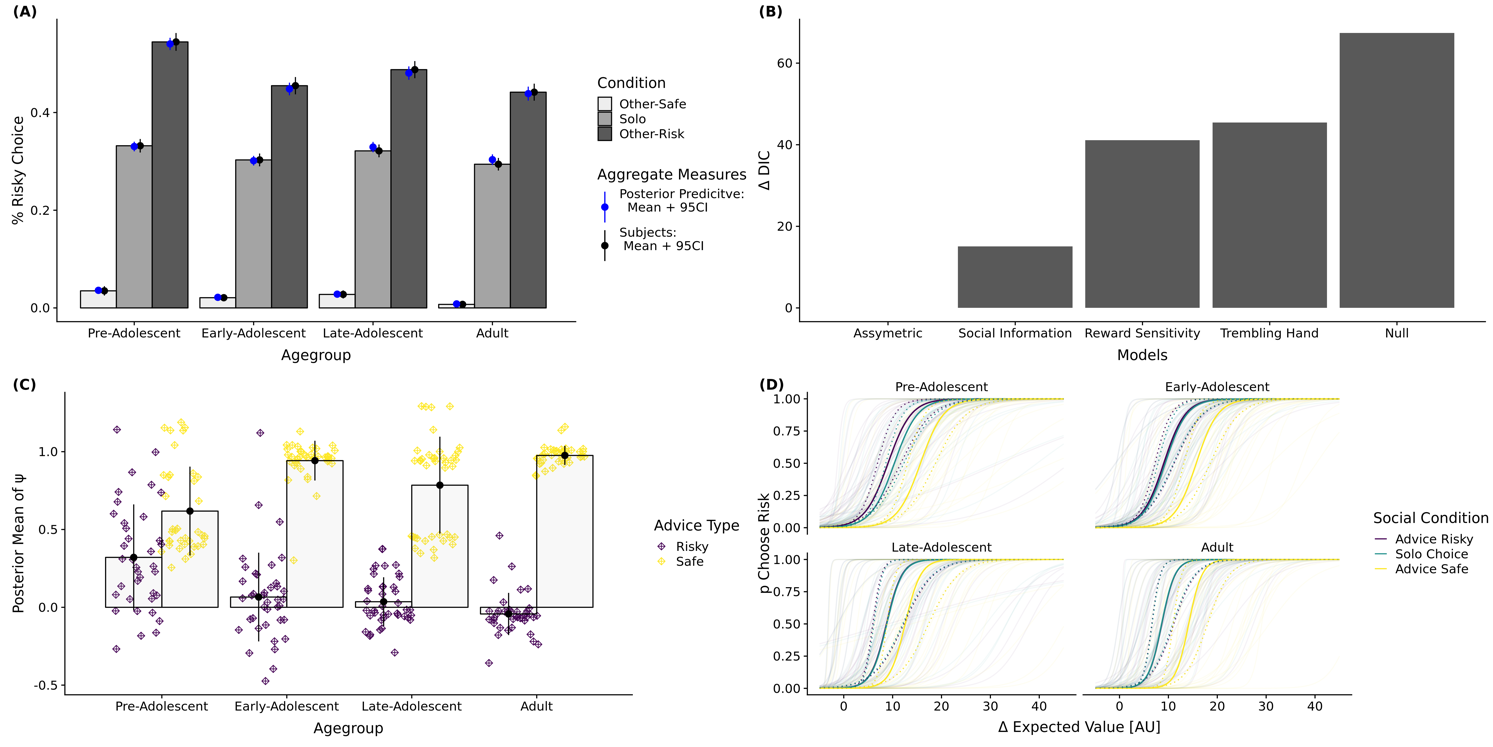


Supplementary Figure 3: Safe and Risky advice, only analyzing risky trials in Blankenstein et al. (2016). (A) Percent risky choice in Blankenstein et al. (2016), by age group and conditions. Black error bars represent the bootstrapped 95% confidence interval. Next to the mean and CI of the subjects’ choices (black), we show simulations under the full posterior from the winning model’ parameter estimates (blue). (B) Difference in DIC fit indices for the whole modelspace, using the winning model as a reference. (C) Posterior Parameter Estimates of risk (purple) and safe (yellow), binned by age group. (D) Predicted probability to choose the risky option given the difference in expected value of the gambles. Colored solid lines correspond to model predictions obtained by computing the mean of subject-level par­ameters in each age group. Colored dashed lines denote upper and lower confidence bounds obtained by computing the standard error of the posterior mean. Transparent lines refer to subject-level predictions.

# Analyzing only Risky Trials in Braams et al 2019.

Subsetting the data in order to only analyze risk trials, yielded that the asymmetric advice model was best suited for the data. However, this procedure resulted in only 36 data points per subject therefore the regression results we report here can likely not be interpreted on a reasonable scale and we just report them here for completness. We find that most participants put higher weight on safety promoting social information (Figure 5C). To judge the statistical relevance of age this pattern, we performed Bayesian general linear regressions, again using age and quadratic age as predictor while treating Ψ_risk_ and Ψ_safe_ as separate dependent variables. We found that linear age was not a good predictor for using risky (β_AgeLin_ = 0.0, CI = [-0.1,0.1]) nor safe advice (β _AgeLin_= -0.1 CI = [-0.1,0.2]). Also, quadratic age trends were not substantial for both risky (β _AgeQuad_= 0.0 CI = [-0.1,0.2]) and or safe advice (β _AgeQuad_= -0.1 CI = [-0.2,0.0]). In sum we find again that safe social information has a greater impact on choice than risky information, model comparison provides evidence that participants assign weight to risky and safe social information differentially.

Supplementary Figure 5: Safe and Risky advice, accounting for the ambiguity condition of Braams et al. (2019).
